# Clinical significance of urinary sodium measurement for predicting pulmonary decongestion: a prospective observational study

**DOI:** 10.1007/s00380-026-02667-2

**Published:** 2026-02-24

**Authors:** Mitsutoshi Oguri, Takahiro Okumura, Satoshi Kojima, Soichiro Maeda, Satoya Yoshida, Yuichiro Koyama, Tomoya Ishiguro, Yusuke Fujikawa, Hiroshi Takahashi, Toyoaki Murohara, Hideki Ishii

**Affiliations:** 1 Department of Cardiology, Kasugai Municipal Hospital, 1-1-1 Takaki-cho, Kasugai, Aichi 486-8510 Japan; 2 Department of Cardiology, Nagoya University Graduate School of Medicine, Nagoya, Japan; 3 Division of Medical Statistics, Fujita Health University, Toyoake, Japan; 4 Department of Cardiovascular Medicine, Gunma University, Maebashi, Japan

**Keywords:** Heart failure, Urinary sodium, Diuretics, Congestion

## Abstract

Diuretic therapy based on the urinary sodium concentration (UNa) is recommended for patients with acute decompensated heart failure (HF). This prospective study evaluated the clinical impact of the UNa on pulmonary decongestion. A total of 450 patients diagnosed with acute decompensated HF who required intravenous furosemide were enrolled. Diuretic therapy was guided by an algorithm based on the UNa measured 1 h after intravenous furosemide administration. Radiographic signs of congestion were compared between patients with a UNa of < 70 mEq/L (*n* = 42) and patients with a UNa of ≥ 70 mEq/L (*n* = 408) using the congestion score index (CSI) measured on days 1 and 3. The change in CSI was smaller in the UNa < 70 mEq/L group than in the UNa ≥ 70 mEq/L group. And the median improvement in CSI in the UNa < 70 mEq/L group was significantly smaller than that in the UNa ≥ 70 mEq/L group. Multivariable logistic regression analysis demonstrated that the serum sodium and C-reactive protein levels and preadmission beta-blocker use were significantly associated with a UNa of < 70 mEq/L. An analysis stratified by the median value (20%) of CSI improvement demonstrated that patients with a < 20% improvement in CSI had higher rates of UNa < 70 mEq/L. In the multivariable analysis, in which the UNa was included as a continuous variable, UNa was significantly associated with poor CSI improvement (*P* = 0.047). In a receiver operating characteristic analysis, the optimal cutoff value for distinguishing a 20% improvement in CSI was 103 mEq/L. A UNa measurement 1 h after intravenous furosemide administration may be useful in predicting pulmonary decongestion in patients with acute decompensated HF, however, the optimal cutoff value requires further validation.

**Supplementary Information:**

The online version contains supplementary material available at https://doi.org/10.1007/s00380-026-02667-2.

## Introduction

In patients with acute heart failure (HF) or acutely decompensated chronic HF, congestion in the most common symptom, which results from a volume overload or an abnormal fluid distribution [1–3]. The widespread recognition of the detrimental effects of prolonged congestion on clinical outcomes and length of hospital stay in patients with HF has accelerated efforts to develop effective strategies for rapid congestion relief [4]. The Heart Failure Association of the European Society of Cardiology (ESC) produced guidelines for performing diuretic therapy in hospitalized patients with acute HF based on early and repeated measurements of the spot urinary sodium concentration (UNa) [5].

The ESC guidelines recommend initiating intravenous (IV) diuretics with a starting dose of 20−40 mg furosemide in furosemide naïve patients or 1−2 times the patient’s preadmission daily oral dose. In cases of an insufficient response to initial IV furosemide, indicated by a UNa of < 50−70 mEq/L after 1–2 h and a urinary output of < 100−150 mL/h after 6 h, a double dose of IV furosemide or combination therapy with diuretics acting at different nephron sites, such as thiazides or acetazolamide, is recommended. Recent clinical trials support these recommendations and demonstrate the efficacy of UNa-guided diuretic therapy [6, 7]. In these trials, the early assessment of the UNa predicted the diuretic response, including changes in body weight and urinary output, and assisted in the risk stratification of clinical outcomes in patients with acute HF. Although prior studies have investigated surrogate markers of congestion, few studies have directly examined whether the UNa can predict clinical improvements in pulmonary congestion.

An algorithm for diuretic therapy developed by the Japanese Circulation Society recommends evaluating the diuretic response using the UNa measured 1–2 h after IV furosemide administration, which closely aligns with the previous ESC guidelines [8]. Among other diuretics, the Japanese guideline indicate that tolvaptan can be used in combination with furosemide. However, administration of tolvaptan has not been adopted as a standard treatment for acute decompensated HF in Western countries, owing to negative findings from randomized controlled trials [9–11]. Although tolvaptan use among patients with acute HF is gradually increasing in Japan and its efficacy in alleviating congestion has been demonstrated [12–14], guidelines for acute-phase UNa-guided diuretic management incorporating tolvaptan have not yet been established.

This study aimed to investigate the efficacy of UNa measured 1 h after IV furosemide administration and subsequent UNa-guided diuretic therapy, including tolvaptan, with the hypothesis that this approach can predict congestion relief in clinical practice.

## Materials and methods

### Study population

**Fig. 1:**
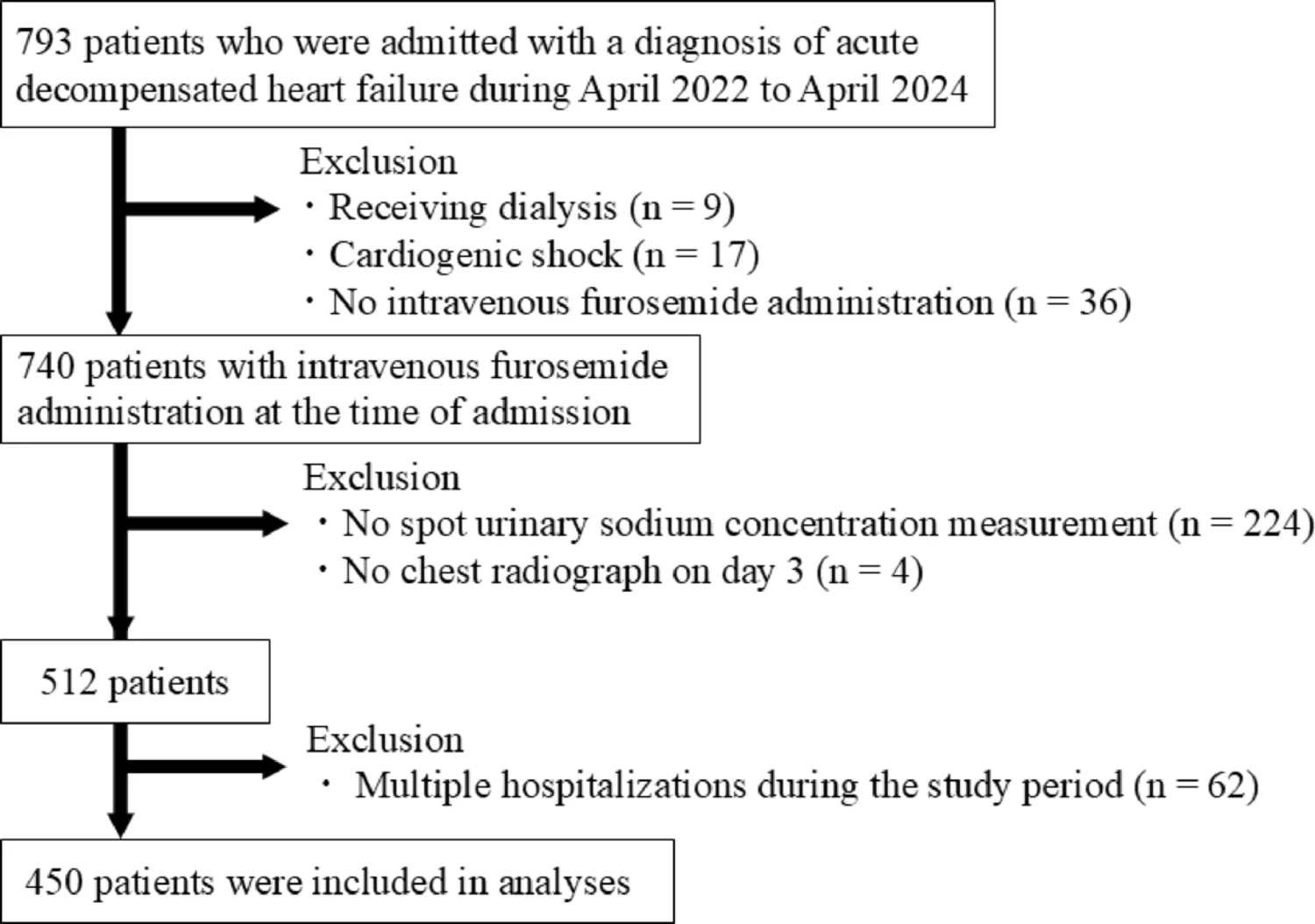
Flowchart of patient enrollment in the study

A two-year recruitment period for this prospective study began in April 2022 at Kasugai Municipal Hospital in Aichi, Japan. The study population consisted of 450 patients admitted with a diagnosis of acute decompensated HF (Fig. 1). Patients on dialysis, those with acute myocardial infarction and/or cardiogenic shock, and those who experienced worsening of HF during hospitalization for other diseases (including surgical procedures and other medical conditions) were excluded because post-admission non-cardiac factors or specialized interventions may affect their prognosis. Patients without UNa measurements at the time of admission were excluded. To reduce the potential confounding from prior treatment effects or clinical events, we included only the first eligible admission per patient during the study period.

The change in congestion score determined from chest radiography was used as the primary endpoint. The secondary endpoints included all-cause death (in-hospital or within one year after discharge) and HF rehospitalization within one year.

### Diuretic management protocol

The differences between our protocol and the international guidelines are as follows: First, we measured the UNa 1 h after the IV furosemide administration. Second, we evaluated the diuretic response using only the UNa. A spot urine sample was collected 1 h after IV furosemide administration either via a bladder catheter or via spontaneous voiding. Based on the UNa measurement, specific options were suggested to enhance the efficacy of acute diuretic therapy. For the patients with UNa ≥ 70 mEq/L (response group), repeated administration of the same IV furosemide dose every 12 h was recommended. In contrast, for the patients with UNa < 70 mEq/L (poor-response group), doubling the furosemide dose or initiating combination therapy with other diuretics (a mineralocorticoid receptor antagonist, tolvaptan, or tolvaptan sodium phosphate) was considered (Supplementary Fig. 1). Compared with other diuretics, thiazides were rarely used in the patients included in this study. The parameters commonly used to differentiate diuretic responses, such as the urinary output and changes in body weight, were insufficient in our setting due to the variability in clinical practice regarding the use of urinary catheters and inconsistent methods of measuring the urinary output or body weight, making it difficult to standardize these metrics in real-world settings. The UNa was used as a guide for configuring the acute-phase diuretic therapy. HF management is based on a comprehensive assessment of subjective symptoms and objective findings.

### Ethics approval

The study protocol complied with the Declaration of Helsinki and was approved by the institutional review board of Kasugai Municipal Hospital (approval number: 495, March 3, 2022) [15]. An opt-out informed consent protocol was used in this study. The consent procedure was reviewed and approved by our institutional ethics committee [15]. None of the participants opted out of the study. This study is registered in the University Hospital Medical Information Network Clinical Trials Registry (UMIN000053797).

### Diagnosis of HF

Acute decompensated HF was diagnosed according to the ESC guidelines based on the clinical signs and symptoms of HF [5]. Each cardiologist made the diagnosis at the time of emergency admission, and the diagnoses were reviewed and verified during departmental conferences.

### Data collection

The clinical characteristics and outcomes of the study participants were assessed via chart review. Vital signs were assessed arrival at the hospital. Most blood samples from the patients were obtained upon hospital admission. The left ventricular ejection fraction (LVEF) was determined using echocardiographic measurements based on the modified biplane Simpson’s method obtained on the day of admission or during hospitalization.

### Radiographic congestion score index

Chest radiography was performed in the emergency department or outpatient clinic at the time of admission. Because most patients experience orthopnea due to worsening HF, the radiographs were obtained with the patients in a sitting position.

The congestion score index (CSI) was used to determine the severity of pulmonary congestion on chest radiography [16]. After dividing each lung field into six zones, the congestion in each zone was evaluated. The degree of congestion was scored as follows: 0, no congestion; 1, cephalization, perivascular/peribronchial cuffing, or Kerley lines; 2, interstitial or localized/mild alveolar pulmonary edema; and 3, intense alveolar pulmonary edema. If more than one-third of the divided fields were obscured by pleural effusion, atelectasis, or cardiac shadows, they were not scored. The CSI was calculated as the sum of the scores divided by the number of available zones. Two cardiologists in our department, who were not blinded to the study protocol, assessed the chest radiograph scores on the day of admission and on day 3.

### Statistical analysis

The statistical analyses were performed using SAS software (version 27; SAS Institute, Inc. Cary, NC, USA). Categorical variables were expressed as the count and percentage and compared between groups using the chi-square test or Fisher’s exact test. Continuous variables were expressed as the median and interquartile range (IQR) or as the mean ± standard deviation, and they were compared using unpaired Student’s t-test (for normally distributed variables by the Shapiro−Wilk test) or the Mann−Whitney *U*-test (for other variables). Univariable and multivariable logistic regression analyses were performed to determine the predictors of a poor response to IV furosemide, and the *P* value, odds ratio (OR), and 95% confidence interval (CI) were calculated. Variables with a *P* value < 0.05 in the univariable analysis were included in the multivariable logistic regression model. Two-way analysis of variance with repeated measures was used to compare the mean differences in within-subject factors between the response and poor-response groups. Further multivariable logistic regression analysis, with poor improvement in CSI as a dependent variable, was performed after adjusting for the variables identified in the univariable analysis. Statistical significance was set at *P* < 0.05.

## Results

### Baseline characteristics and in-hospital treatment

The characteristics of the study participants according to their UNa levels are presented in Table 1. The median age of the study was 83 (IQR: 76–89) years. The median UNa level 1 h after IV furosemide administration was 120 mEq/L (IQR: 100–131 mEq/L). The distribution of UNa was shown in Supplementary Fig. 2. When stratified by the natriuretic response, the patients in the poor-response group tended to have higher baseline levels of C-reactive protein (CRP) and brain natriuretic peptide (BNP) and lower sodium levels, estimated glomerular filtration rates (eGFR), and LVEF (Table 1). The rates of preadmission use of tolvaptan, beta-blockers, and sodium-glucose cotransporter–2 inhibitors were higher in the poor-response group than in the response group. The rate of intensified diuretic therapy, including furosemide dose intensification and/or combination diuretic therapy with tolvaptan, tolvaptan sodium phosphate, potassium canrenoate, was significantly higher in the poor-response group than in the response group (64.3% vs. 39.2%, *P* = 0.002). The clinical factors associated with protocol adherence in the poor-response group were a higher level of BNP [1204 (560–1844) pg/mL vs. 517 (429–855) pg/mL, *P* = 0.035] and a higher rate of preadmission use of tolvaptan (100% vs. 55.9%, *P* = 0.035). The median initial IV furosemide dose at admission and the total IV furosemide dose on day 2 were similar between the two groups. The total IV furosemide dose on day 1 could not be evaluated because of the varying admission times. IV tolvaptan sodium phosphate and inotropes were administered marginally more frequently in the poor-response group than in the response group. The administration rates of other IV drug therapies were similar between the two groups. The length of hospital stay, in-hospital mortality rate, 1-year post-discharge mortality rate, and 1-year post-discharge HF rehospitalization rate were comparable between the two groups (Table 2).

**Table 1:** Characteristics of study participants stratified by UNa levels 1 h after intravenous Furosemide administration

Variable	All patients *n* = 450	UNa < 70 mEq/L *n* = 42	UNa ≥ 70 mEq/L *n* = 408	*P* value
Age (years)	83 (76–89)	81 (75–89)	83 (76–89)	0.701
Male sex (%)	55.3	54.8	55.4	0.938
Current or former smoker (%)	41.3	31.0	42.4	0.333
Body mass index (kg/m^2^)	23.4 ± 4.2	23.6 ± 4.1	23.4 ± 4.2	0.722
Ischemic (%)	33.9	35.7	33.7	0.789
*Comorbidities* (%)				
Dyslipidemia	62.9	71.4	62.1	0.232
Type 2 diabetes mellitus	40.2	50.0	39.2	0.175
Hypertension	72.8	69.1	73.2	0.569
Atrial fibrillation	44.6	35.7	45.6	0.256
Previous myocardial infarction	20.3	28.6	19.5	0.163
Previous stroke	13.4	11.9	13.6	1.000
Chronic obstructive pulmonary disease	4.9	2.4	5.2	0.710
Previous hospitalization for HF (before the study period, %)	30.4	38.1	29.6	0.290
*Initial evaluation*				
Systolic blood pressure (mmHg)	143 (125–163)	133 (120–159)	144 (127–163)	0.061
Diastolic blood pressure (mmHg)	89 (74–104)	82 (65–108)	90 (75–104)	0.226
Heart rate (beat per minute)	96 (80–114)	91 (76–107)	96 (82–115)	0.132
Sodium (mEq/L)	142 (140–145)	141 (136–144)	142 (140–145)	0.027
Potassium (mEq/L)	4.0 (3.6–4.3)	4.1 (3.3–4.6)	4.0 (3.6–4.3)	0.981
eGFR (mL/min/1.73m^2^)	42.1 (30.2–55.8)	37.7 (21.8–48.3)	42.5 (30.5–56.4)	0.036
Albumin (mg/dL)	3.2 ± 0.5	3.1 ± 0.4	3.2 ± 0.5	0.214
Total bilirubin (mg/dL)	0.9 (0.6–1.3)	0.8 (0.6–1.4)	0.9 (0.6–1.3)	0.559
CRP (mg/L)	7.6 (2.3–20.6)	13.3 (5.8–42.2)	7.1 (2.2–19.3)	0.013
BNP (pg/mL)	540 (287–1014)	759 (473–1624)	514 (269–971)	0.003
Hemoglobin (mg/dL)	11.3 ± 2.5	11.1 ± 2.5	11.3 ± 2.5	0.728
*NYHA functional class* (%)				0.331
II	3.6	0	4.0	
III	29.8	26.2	30.1	
IV	66.6	73.8	65.9	
LVEF^a^ (%)	51 (38–64)	41 (31–63)	52 (38–64)	0.036
UNa 1 h after intravenous furosemide administration (mEq/L)	120 (100–131)	51 (32–60)	122 (107–132)	< 0.001
Congestion score index				
Day 1 (at admission) (pts)	2.2 (1.8–2.5)	2.0 (1.8–2.4)	2.2 (1.8–2.5)	0.843
Day 3 (pts)	1.7 (1.3–2.2)	1.8 (1.3–2.3)	1.7 (1.3–2.0)	0.184
Improvement rate from days 1 to 3 (%)	20.0 (7.1–33.3)	12.9 (0–30.1)	20.0 (7.7–33.3)	0.048
*Preadmission medications*				
Loop diuretics (%)	43.3	40.5	43.6	0.746
Furosemide-equivalent dose (mg)	0 (0–20)	0 (0–20)	0 (0–20)	0.939
Tolvaptan (%)	8.5	19.1	7.4	0.018
Renin angiotensin system inhibitors (%)	39.8	35.7	40.2	0.622
Beta-blockers (%)	33.8	57.1	31.4	0.002
Mineralocorticoid receptor blockers (%)	19.1	23.8	18.6	0.413
Sodium-glucose cotransporter–2 inhibitors (%)	10.0	22.5	8.7	0.011
*In-hospital drug therapy*				
Intensification of furosemide dose and/or combination diuretic therapy	41.6	64.3	39.2	0.002
Intensification of furosemide dose (%)	19.1	35.7	17.4	0.007
Combination diuretic therapy (%)	30.2	47.6	28.4	0.010
Initial furosemide dose at admission (mg)	20 (20–20)	20 (20–20)	20 (20–20)	0.328
Total furosemide dose at day 2 (mg)	40 (20–40)	40 (20–80)	40 (20–40)	0.057
Tolvaptan sodium phosphate (%)	8.7	19.1	7.6	0.020
Potassium canrenoate (%)	6.9	7.1	6.9	1.000
Carperitide (%)	7.3	2.4	7.8	0.346
Vasodilators (%)	32.0	26.2	32.6	0.488
Inotropes (%)	9.8	26.2	8.1	0.001
Non-invasive ventilations (%)	6.9	7.1	6.9	1.000
Tolvaptan (newly administration, %)	13.1	14.3	13.0	0.810

Data are presented as means ± standard deviation, median (interquartile range), or percentage (%) unless otherwise specified

UNa, urinary sodium concentration; HF, heart failure; eGFR, estimated glomerular filtration rate; CRP, C-reactive protein; BNP, B-type natriuretic peptide; NYHA, New York Heart Association; LVEF, left ventricular ejection fraction

^a^LVEF was either known on the day of admission or measured during hospitalization

**Table 2:** Clinical outcomes stratified by UNa levels 1 h after intravenous Furosemide administration

Variable	All patients *n* = 450	UNa < 70 mEq/L *n* = 42	UNa ≥ 70 mEq/L *n* = 408	*P* value
Length of hospital stay (days)	13 (9–19)	14 (10–27)	13 (9–18)	0.471
In-hospital death (%)	4.7	9.5	4.2	0.122
1-year post-discharge death (%)	10.7	18.4	10.0	0.163
1-year post-discharge HF rehospitalization (%)	16.6	18.4	16.4	0.819

Data are presented as means ± standard deviation, median (interquartile range), or percentage (%) unless otherwise specified

UNa, urinary sodium concentration; HF, heart failure

**Fig. 2:**
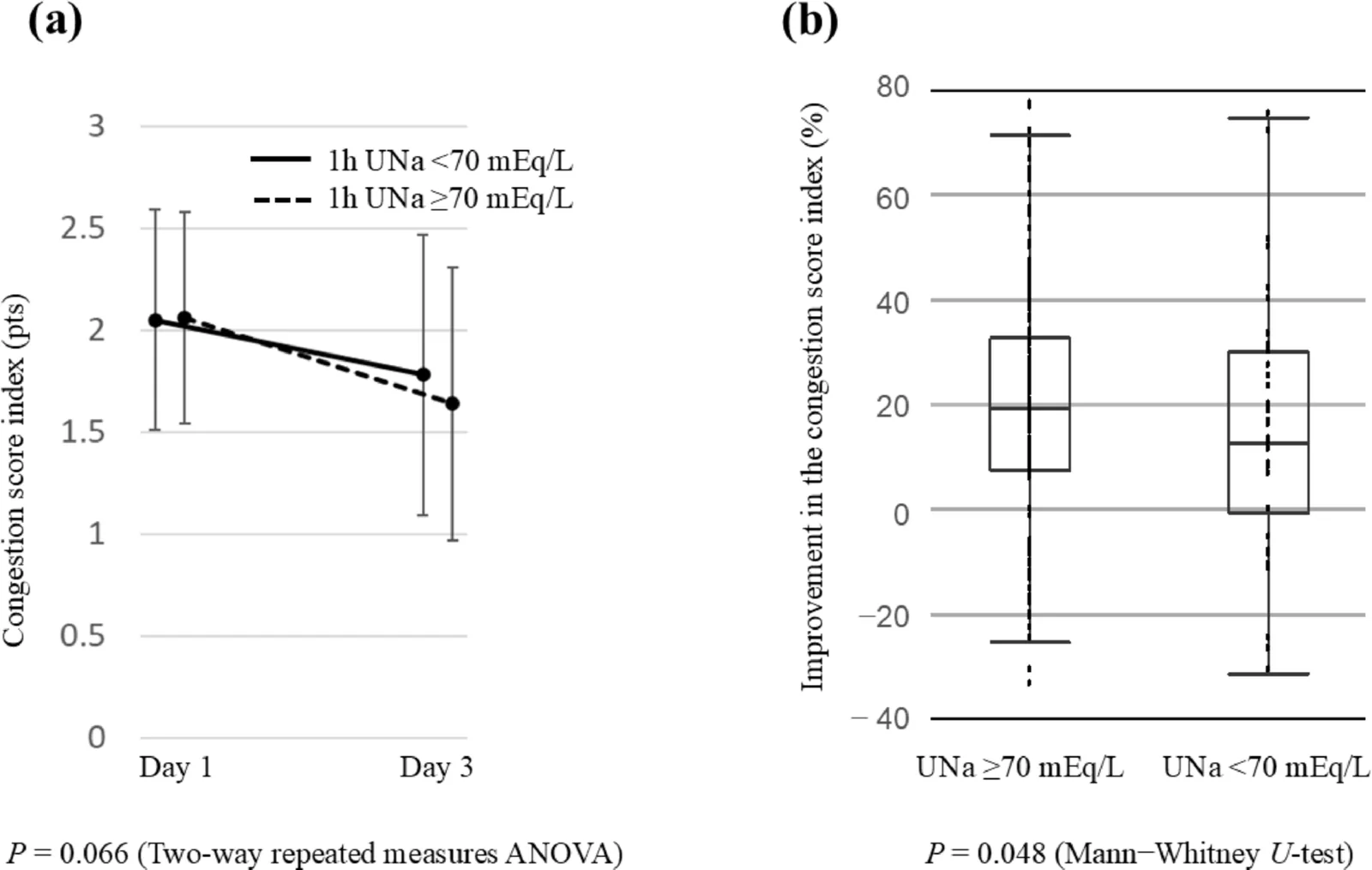
**a** Changes in the congestion score index between days 1 and 3 using a urinary sodium concentration (UNa) cutoff value of 70 mEq/L. **b** Improvement in the congestion score index from days 1 to 3 in patients with a UNa of < 70 mEq/L and in those with a UNa of ≥ 70 mEq/L. The data are expressed as the mean ± standard deviation. ANOVA, analysis of variance

The median CSI on day 1 and that on day 3 were comparable between the groups. In the poor-response group, the CSI changed from 2.0 (1.8−2.4) to 1.8 (1.3−2.3), whereas in the response group, it changed from 2.2 (1.8–2.5) to 1.7 (1.3–2.0), indicating that the magnitude of the change tended to be smaller in the poor-response group, but the difference was not statistically significant (*P* = 0.066). In contrast, the improvement rate from days 1 to 3 was significantly smaller in the poor-response group (12.9% [IQR: 0–30.1%] vs. 20.0% [IQR: 7.7–33.3%], *P* = 0.048) (Table 1; Fig. 2).

### Analysis of factors associated with a poor response to IV Furosemide

Univariable logistic regression analysis revealed that the sodium level, eGFR, CRP level, BNP level, LVEF, and preadmission use of tolvaptan, beta-blockers, and sodium-glucose cotransporter–2 inhibitors were significantly associated with a poor response to IV furosemide. In the multivariable logistic regression analysis, the sodium level was inversely associated with a poor response to IV furosemide (OR = 0.90, 95% CI: 0.83–0.97, *P* = 0.005), whereas the CRP level and preadmission use of beta-blockers were positively associated with a poor response (OR = 1.14, 95% CI: 1.05–1.24, *P* = 0.002; OR = 2.64, 95% CI: 1.25–5.56, *P* = 0.011) (Table 3).

**Table 3:** Univariable and multivariable logistic regression analysis of factors associated with a poor response to intravenous Furosemide

Characteristics	Univariable		Multivariable	
	OR (95%CI)	*P* value	OR (95%CI)	*P* value
Sodium	0.89 (0.83–0.95)	< 0.001	0.90 (0.83–0.97)	0.005
eGFR	0.98 (0.96–1.00)	0.040	0.99 (0.97–1.01)	0.185
CRP	1.12 (1.04–1.20)	0.002	1.14 (1.05–1.24)	0.002
BNP	1.00 (1.00–1.00)	0.026	1.00 (1.00–1.00)	0.267
LVEF	0.98 (0.96–1.00)	0.037	0.98 (0.96–1.00)	0.089
Preadmission tolvaptan use	2.93 (1.25–6.90)	0.014	2.21 (0.79–6.23)	0.132
Preadmission beta-blockers use	2.92 (1.53–5.56)	0.001	2.64 (1.25–5.56)	0.011
Preadmission sodium-glucose cotransporter–2 inhibitors use	3.04 (1.34–6.91)	0.008	2.20 (0.88–5.53)	0.092

UNa, urinary sodium concentration; eGFR, estimated glomerular filtration rate; CRP, C reactive protein; BNP, B-type natriuretic peptide; LVEF, left ventricular ejection fraction; OR, odds ratio; CI, confidence interval

### Analysis of factors associated with poor CSI improvement

**Fig. 3:**
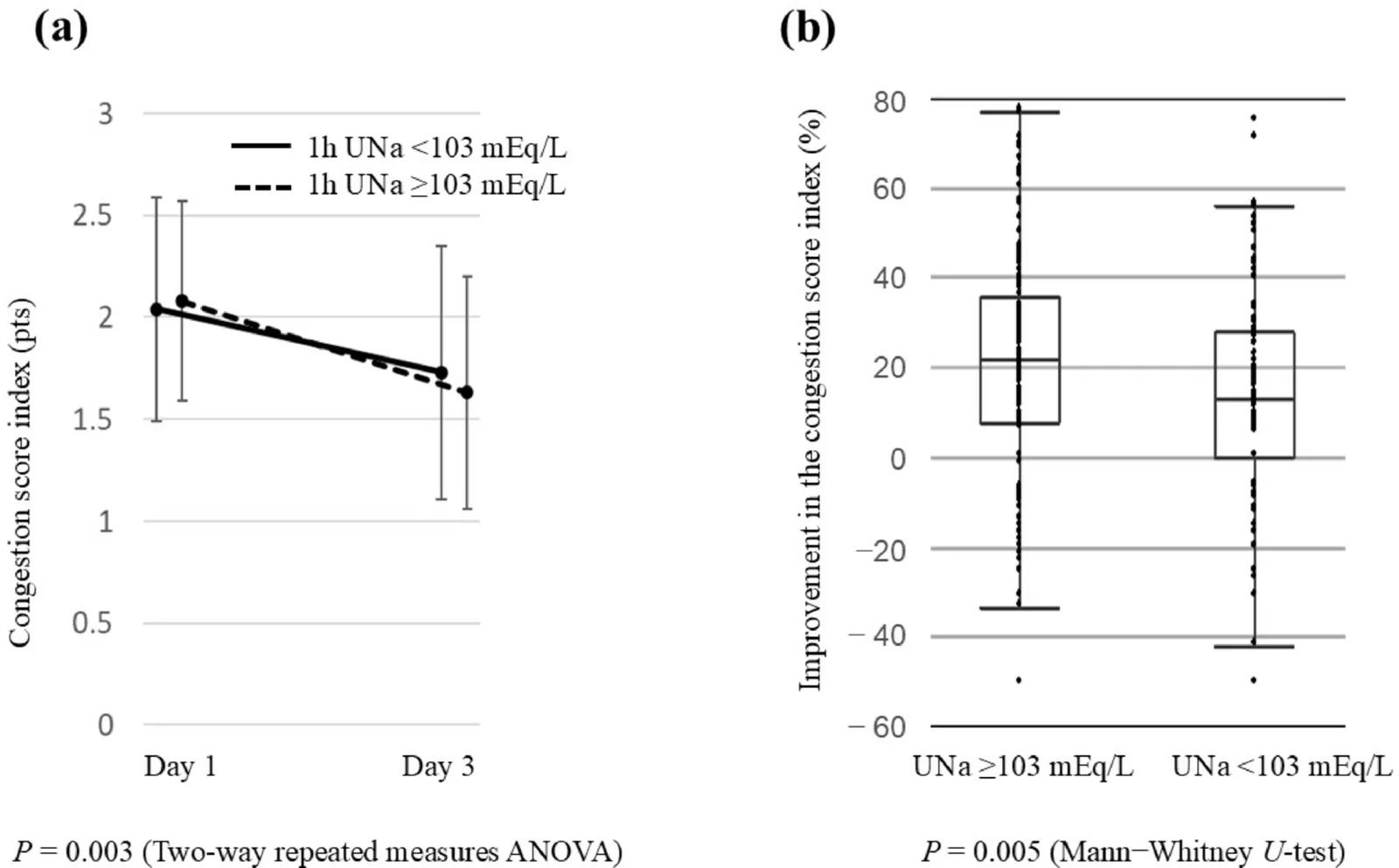
**a** Changes in the congestion score index between days 1 and 3 using a urinary sodium concentration (UNa) cutoff value of UNa 103 mEq/L. **b** Improvement in the congestion score index from days 1 to 3 in patients with a UNa of < 103 mEq/L and in those with a UNa of ≥ 103 mEq/L. The data are expressed as the mean ± standard deviation. ANOVA, analysis of variance

Next, we analyzed the association between a poor response to furosemide and CSI improvement rate on chest radiographs, stratified by median value of 20%. Patients with < 20% improvement had significantly lower systolic and diastolic blood pressures, heart rates, albumin levels, and in-hospital IV administration of vasodilators. In contrast, they were significantly older and had higher rates of UNa < 70 mEq/L 1 h after IV furosemide administration, and preadmission loop diuretic use, and in-hospital IV administration of tolvaptan sodium phosphate and inotropes compared with those with ≥ 20% improvement (Table 4). Additionally, the length of hospital stay, in-hospital mortality rate, 1-year post-discharge mortality rate were significantly higher in patients with a < 20% improvement in CSI (Table 5). Univariable logistic regression analysis demonstrated that the systolic blood pressure, heart rate, albumin level, UNa 1 h after IV furosemide administration (both as a continuous variable and dichotomized variable, with a cutoff value of < 70 mEq/L), preadmission loop diuretic use, and in-hospital IV administration of tolvaptan sodium phosphate, vasodilators, and inotropes were significantly associated with poor CSI improvement (*P* < 0.05). In the multivariable analysis, which adjusted for variables identified in the univariable analysis, a UNa of < 70 mEq/L was not associated with poor CSI improvement. However, the UNa as a continuous variable was significantly associated with poor CSI improvement (OR for per 5 mEq/L decrease = 1.05, 95% CI: 1.00–1.05, *P* = 0.047). Therefore, we performed a receiver operating characteristic analysis, which indicated that the optimal cutoff value for distinguishing a 20% improvement in CSI was 103 mEq/L. Further multivariable logistic regression analysis demonstrated that a UNa of < 103 mEq/L was significantly and independently associated with poor CSI improvement (OR = 1.63, 95% CI: 1.03–2.58, *P* = 0.037) (Table 6). The patients with a UNa of < 103 mEq/L exhibited a change in the CSI from 2.0 (1.7–2.5) to 1.7 (1.3–2.2), whereas those with a UNa ≥ 103 mEq/L exhibited a change from 2.2 (1.8–2.5) to 1.7 (1.3–2.0). The change in the CSI was significantly smaller in patients with a UNa of < 103 mEq/L (*P* = 0.003). The improvement rate from days 1 to 3 was also significantly smaller in the patients with a UNa of < 103 mEq/L (13.8% [IQR: 0–28.2%] vs. 22.2% [IQR: 8.0–35.7%], *P* = 0.005) (Fig. 3). Additionally, in-hospital mortality rate (9.1% vs. 2.7%, *P* = 0.008), 1-year post-discharge HF rehospitalization rate (22.7% vs. 14.4%, *P* = 0.043) were significantly higher in the patients with a UNa of < 103 mEq/L.

**Table 4:** Characteristics of study participants stratified by the rate of CSI improvement from days 1 to 3

Variable	All patients *n* = 450	Improvement in CSI < 20% *n* = 218	Improvement in CSI ≥ 20% *n* = 232	*P* value
Age (years)	83 (76–89)	85 (77–89)	82 (75–88)	0.023
Male sex (%)	55.3	54.1	56.5	0.618
Current or former smoker (%)	41.3	37.2	45.3	0.127
Body mass index (kg/m^2^)	23.4 ± 4.2	23.3 ± 4.1	23.4 ± 4.3	0.841
Ischemic (%)	33.9	30.2	37.2	0.119
*Comorbidities* (%)				
Dyslipidemia	62.9	63.1	62.8	0.937
Type 2 diabetes mellitus	40.2	37.2	43.1	0.199
Hypertension	72.8	73.3	72.3	0.816
Atrial fibrillation	44.6	48.9	40.7	0.083
Previous myocardial infarction	20.3	18.4	22.1	0.338
Previous stroke	13.4	10.7	16.0	0.096
Chronic obstructive pulmonary disease	4.9	3.7	6.1	0.280
Previous hospitalization for HF (before the study period, %)	30.4	31.8	29.0	0.521
*Initial evaluation*				
Systolic blood pressure (mmHg)	143 (125–163)	139 (121–156)	148 (132–168)	< 0.001
Diastolic blood pressure (mmHg)	89 (74–104)	87 (72–101)	92 (75–108)	0.008
Heart rate (beat per minute)	96 (80–114)	91 (76–110)	100 (85–117)	0.002
Sodium (mEq/L)	142 (140–145)	142 (140–145)	142 (140–144)	0.644
Potassium (mEq/L)	4.0 (3.6–4.3)	4.0 (3.6–4.3)	4.0 (3.6–4.3)	0.418
eGFR (mL/min/1.73m^2^)	42.1 (30.2–55.8)	41.3 (29.1–54.4)	43.8 (31.5–58.4)	0.355
Albumin (mg/dL)	3.2 ± 0.5	3.1 ± 0.5	3.3 ± 0.5	< 0.001
Total bilirubin (mg/dL)	0.9 (0.6–1.3)	0.9 (0.6–1.4)	0.9 (0.6–1.2)	0.471
CRP (mg/L)	7.6 (2.3–20.6)	7.7 (2.4–28.8)	7.1 (2.2–17.6)	0.197
BNP (pg/mL)	540 (287–1014)	572 (286–1090)	516 (287–959)	0.459
Hemoglobin (mg/dL)	11.3 ± 2.5	11.2 ± 2.5	11.3 ± 2.5	0.848
*NYHA functional class* (%)				0.184
II	3.6	5.1	2.2	
III	29.8	31.0	28.6	
IV	66.6	63.9	69.3	
LVEF^a^ (%)	51 (38–64)	53 (38–64)	49 (38–64)	0.390
UNa 1 h after intravenous furosemide administration (mEq/L)	120 (100–131)	116 (91–129)	122 (105–134)	0.002
UNa 1 h after intravenous furosemide administration < 70 mEq/L (%)	9.3	12.4	6.5	0.031
*Preadmission medications* (%)				
Loop diuretics	43.3	48.6	38.4	0.028
Tolvaptan	8.5	8.8	8.2	0.817
Renin angiotensin system inhibitors	39.8	37.2	42.2	0.271
Beta-blockers	33.8	36.2	31.5	0.285
Mineralocorticoid receptor blockers	19.1	21.6	16.8	0.200
Sodium-glucose cotransporter–2 inhibitors	10.0	9.9	10.0	0.948
*In-hospital intravenous drug therapy*				
Initial furosemide dose at admission (mg)	20 (20–20)	20 (20–20)	20 (20–20)	0.475
Total furosemide dose at day 2 (mg)	40 (20–40)	40 (20–40)	40 (20–40)	0.942
Tolvaptan sodium phosphate (%)	8.7	12.8	4.7	0.002
Potassium canrenoate (%)	6.9	6.4	7.3	0.715
Carperitide (%)	7.3	7.8	6.9	0.722
Vasodilators (%)	32.0	27.1	36.6	0.030
Inotropes (%)	9.8	13.3	6.5	0.017
Non-invasive ventilations (%)	6.9	8.3	5.6	0.352
Tolvaptan (newly administration, %)	13.1	16.1	10.3	0.073

Data are presented as means ± standard deviation, median (interquartile range), or percentage (%) unless otherwise specified

CSI, congestion score index; HF, heart failure; eGFR, estimated glomerular filtration rate; CRP, C-reactive protein; BNP, B-type natriuretic peptide; NYHA, New York Heart Association; LVEF, left ventricular ejection fraction; UNa, urinary sodium concentration

^a^LVEF was either known on the day of admission or measured during hospitalization

**Table 5:** Clinical outcomes stratified by the rate of CSI improvement from days 1 to 3

Variable	All patients *n* = 450	Improvement in CSI < 20% *n* = 218	Improvement in CSI ≥ 20% *n* = 232	*P* value
Length of hospital stay (days)	13 (9–19)	14 (10–21)	12 (9–17)	0.003
In-hospital death (%)	4.7	6.9	2.6	0.043
1-year post-discharge death (%)	10.7	14.3	7.5	0.028
1-year post-discharge HF rehospitalization (%)	16.6	16.8	16.4	0.916

Data are presented as median (interquartile range) or percentage (%)

CSI, congestion score index; HF, heart failure

**Table 6:** Univariable and multivariable logistic regression analysis associated with poor CSI improvement

Characteristics	Univariable			Multivariable	
	OR (95%CI)	*P* value		OR (95%CI)	*P* value
UNa 1 h after intravenous furosemide administration (continuous) (per 5 mEq/L decrease)	1.05 (1.00–1.11)	0.001		1.05 (1.00–1.05)	0.047
< 70 mEq/L	2.15 (1.06–3.96)	0.034		1.57 (0.78–3.12)	0.210
< 103 mEq/L	1.93 (1.26–2.95)	0.002		1.63 (1.03–2.58)	0.037

Multivariable logistic regression analysis was performed with adjusted for systolic blood pressure, heart rate, albumin level, preadmission loop diuretic use, in-hospital intravenous administration of tolvaptan sodium phosphate, vasodilators, and inotropes. UNa, urinary sodium concentration; CSI, congestion score index; OR, odds ratio; CI, confidence interval

## Discussion

A major strength of this study is its novel findings. A UNa measurement 1 h after IV furosemide administration may be useful in predicting pulmonary decongestion, however, the optimal cutoff value requires further validation. Our finding that early UNa measurement correlates with pulmonary congestion and highlight the need for further studies to determine optimal cutoff values for clinical decision-making. Another strength is the ability to identify patient characteristics with a poor natriuretic response during the early course of acute HF treatment.

Although prior studies have examined the relationship between UNa measurement and surrogate makers such as body weight change, urine volume, and clinical outcomes, the specific association between natriuretic response at 1 h after IV furosemide administration and objective radiographic decongestion remains to be elucidated. Therefore, our study focused on radiographic pulmonary congestion, assessed using the CSI, which is a validated, semi-quantitative, and reproducible method [16]. The CSI is correlated with clinical outcomes and offers a strong diagnostic performance. We recognize that the CSI primarily reflects pulmonary congestion and may not reflect all aspects of systemic fluid overload, and we believe that its objective nature provides a robust assessment of congestion dynamics during acute HF management. The primary endpoint of our study included the changes and improvements in pulmonary congestion, which are important and actionable therapeutic targets in acute settings. The Efficacy of Standardized Diuretic Protocol in Acute Heart Failure (ENACT-HF) trial demonstrated the efficacy of a natriuresis-guided diuretic protocol in terms of short-term diuresis and length of hospital stay [7]. The study also evaluated the congestion score, which includes peripheral edema, pleural effusion, and ascites, primarily reflecting fluid overload. It does not incorporate other modalities, such as radiography; therefore, pulmonary congestion was not assessed. Although comprehensive congestion scores exist, chest radiography is performed in almost all cases with HF and can be evaluated rapidly, therefore, we assessed CSI for the evaluation of pulmonary congestion. However, future studies that integrate pulmonary and systemic congestion are required.

Our study suggests that measuring UNa may help identify patients with a poor natriuretic response who could benefit from the intensification of furosemide dosage and combination therapy with other diuretics. Based on our analysis of the predictors of poor response to furosemide (UNa < 70 mEq/L), patients with low sodium levels, elevated CRP levels, and preadmission use of beta-blockers, potentially indicating more severe HF, may be a target for furosemide dose intensification and/or combination diuretic therapy from the time of hospital admission. Our results are partially consistent with those of the previous studies. Luk A et al. showed that patients with poor natriuretic response, using UNa measured 1 h after IV furosemide with a cutoff value of < 60 mEq/L, were more likely to have low sodium levels and blood pressure, less preadmission use of angiotensin-converting-enzyme inhibitor/angiotensin II receptor and beta-blockers [17]. In our study, and that preadmission use of beta-blockers was a significant predictor of poor response to furosemide, which was opposite to their finding. Given that preadmission beta-blocker use was associated with eGFR and BNP levels as risk factors for poor natriuresis, this relationship may be clinically insignificant. Despite the limited number of studies on preadmission medications and natriuresis, other reports have indicated no association between beta-blockers and the degree of natriuresis [18, 19]. Although we identified CRP levels as a significant factor for poor response to furosemide, this value has not been included in previous studies [17, 19, 20].

To identify more patients with poor natriuresis, we defined a cutoff value for UNa at 70 mEq/L, representing the upper limit of the range recommended by the ESC guidelines [5]. Previous studies have shown that lower UNa levels are significantly associated with the diuretic resistance [20, 21], a longer hospital stay [18], and an increased mortality [19, 22], although the timing of the UNa measurement and the UNa cutoff values varied across studies. Because UNa can be influenced by sodium and water intake and urine concentration [23], hours after IV furosemide administration may be the optimal time for measurement. Collins et al. conducted the first prospective study assessing the early detection of diuretic resistance using UNa 1 h after IV furosemide administration in patients presenting to the emergency department [24]. The median UNa in their study was 71 mEq/L, and the cutoff for diuretic resistance was 35.4 mEq/L, both of which were lower than those in our study, possibly because of the inclusion of patients with more severe conditions, such as higher BNP levels. Their study is notable from the viewpoint of prompt identification of patients who require early intensification of diuretic treatment; however, the small sample size, younger age, and lack of a detailed algorithm based on UNa make it difficult to adapt to current clinical practice. After the publication of the algorithm from the Heart Failure Association of the ESC, the Pragmatic Urinary Sodium-based algorithms in the Acute Heart Failure (PUSH-AHF) trial [6] and ENACT-HF trial [7] were performed, and it was reported that natriuresis-guided management could be safe and suitable for all clinical situations. These mean UNa 2 h after IV furosemide administration in the PUSH-AHF trial was 94 ± 32 mEq/L, and that in the ENACT-HF trial was 96 ± 28 mEq/L. Despite the difference in measurement timing (1 h vs. 2 h after IV furosemide administration), these values are lower than those reported in our study. Higher N-terminal pro-BNP levels and a lower LVEF, as observed in these two studies, may result lower UNa levels following IV furosemide administration. The proportion of patients with poor natriuresis was 29.0% in the PUSH-AHF trial (cutoff value: <70 mEq/L) and 12.6% in the ENACT-HF trial (cutoff value: <50 mEq/L). In contrast, only 9.3% of patients in our study had poor natriuresis. Based on these facts, the ideal cutoff value of the UNa for the Japanese population may be slightly higher than that in the international guidelines. Therefore, the UNa cutoff value of 103 mEq/L, obtained from the analysis of clinical congestion improvement in the present study may be plausible. Although the analysis using the UNa cutoff value of 70 mEq/L did not independently predict CSI improvement in the multivariable analysis, the continuous association between UNa and pulmonary congestion improvement remains clinically meaningful and complementary information. Therefore, we presented the exploratory continuous analysis in addition to the primary hypothesis-based analysis using the UNa cutoff value of 70 mEq/L in the present study. We believe this approach, presenting both analyses as hypothesis-generating, provides a more comprehensive interpretation. Although there was no difference in mortality when using the UNa cutoff value of 70 mEq/L, significant differences in in-hospital mortality and 1-year post-discharge HF rehospitalization rate were observed when the exploratory cutoff value of 103 mEq/L was applied. Given that a cutoff value of UNa 103 mEq/L was derived through post-hoc analysis rather than being pre-specified, this cutoff should be interpreted with caution and may enable a clearer prediction of pulmonary congestion improvement, pending validation in independent cohorts. In addition, optimal covariate selection for this cutoff value may differ in the multivariable analysis. These findings suggest that UNa may serve as a prognostic indicator, but prospective validation is still warranted.

Administration of tolvaptan and tolvaptan sodium phosphate have been approved in Japan for patients with acute decompensated HF and furosemide resistance, whereas inotropes are indicated for patients with hypoperfusion, as per national guidelines [8]. The results suggest that more frequent use of IV tolvaptan sodium phosphate and inotropes in the poor response group may be appropriate. However, as there was no relationship between tolvaptan sodium phosphate and inotropes and improvement in congestion itself, it cannot be confirmed that these agents may have contributed to similar congestion relief or equivalent in-hospital outcomes.

This study has some limitations. First, not all participants underwent diuretic adjustment in accordance with the study recommendation, which may be attributed to physician discretion or the potential influence of combination therapy with non-diuretic agents. The moderate level of protocol adherence reflects the pragmatic nature of the trial; however, a randomized controlled trial would be necessary. Second, it was conducted at a single center and may not have been validated in other institutions. Third, the UNa was not measured in all eligible patients, precluding consecutive enrollments. Fourth, correlations with other natriuresis markers and combinations with other clinical parameters were not assessed. Furthermore, an analysis stratified by factors that could affect UNa levels, such as sodium-glucose cotransporter–2 inhibitors use or the degree of renal dysfunction, was not performed. Fifth, the cardiologists assessing the CSI were not blinded to the study protocol. Fifth, the UNa cutoff value of 103 mEq/L was derived from post-hoc receiver operating characteristic analysis and requires validation in independent cohorts.

In conclusion, a UNa measurement 1 h after IV furosemide administration may be useful for forecasting pulmonary decongestion in patients with acute decompensated HF. However, further studies are needed to establish optimal cutoff values for clinical application.

## Electronic Supplementary Material

Below is the link to the electronic supplementary material.

**Supplementary Figure 1**. Urinary sodium concentration-guided diuretic management protocol.

**Supplementary Figure 2**. Distribution of urinary sodium concentration measured 1 h after intravenous furosemide administration.
